# Branchial Cleft-Like Cysts Involving 3 Different Organs: Thyroid Gland, Thymus, and Parotid Gland

**DOI:** 10.1097/MD.0000000000001758

**Published:** 2015-10-23

**Authors:** Tadao Nakazawa, Tetsuo Kondo, Naoki Oishi, Ippei Tahara, Kazunari Kasai, Tomohiro Inoue, Kunio Mochizuki, Ryohei Katoh

**Affiliations:** From the Department of Pathology, Interdisciplinary Graduate School of Medicine and Engineering, University of Yamanashi, Yamanashi, Japan.

## Abstract

Branchial cleft cysts (BCCs) are also named lateral cervical cysts and widely acknowledged as being derived from embryonic remnants. Lymphoepithelial cysts (LECs) generally show microscopic features that are identical to those of BCCs, and rarely occur at unusual sites or organs.

A case of multiple cysts arising in both lobes of the thyroid gland, thymus, and right parotid gland in a 41-year-old man is reported. Clinically, the patient presented with Hashimoto's thyroiditis for about 20 years and had past histories of idiopathic thrombocytopenic purpura and severe respiratory infection.

This case is unusual in that multiple cysts arose synchronously and/or heterochronously and grew, increasing their sizes in these different organs. Microscopic examinations revealed that all of the cysts were composed of squamous epithelium, dense lymphoid tissue with germinal centers, and a fibrous capsule. These findings corresponded to those of BCCs or LECs. It is notable that the histopathological features were nearly the same in the individual organs. A review of the literature disclosed no previous such reported cases.

The etiology is unknown. However, based upon the similar histopathological features of all the excised specimens, common immune and/or hematopoietic disorders may have contributed to their occurrence and development in association with putative genetic abnormalities.

## INTRODUCTION

Branchial cleft cysts (BCCs), also called lateral cervical cysts, occur mostly in the lateral cervical area anterior to the sternocleidomastoid muscle. Microscopically, the cyst is usually lined by squamous or squamoid epithelium, occasionally accompanied by ciliated columnar epithelium and mucous cells. The cyst wall contains abundant lymphoid tissues with germinal centers.^1,2^ It is widely acknowledged that the lesions are derived from the embryonic remnants of the branchial pouch. The lesions result from incomplete obliteration of the branchial arches that remain dormant until they are stimulated to display cystic growth later in life.

BCCs have often been referred to using the descriptive diagnosis “lymphoepithelial cysts (LECs).” LECs and BCCs show similar microscopic features and are indistinguishable from each other. LCCs have been reported to occur in unusual sites or organs, such as the oral cavity,^3^ parotid gland,^4^ pancreas,^5^ and thyroid gland.^6,7^ On the basis of morphological similarity, embryonic rests have also been considered to be among the candidates for the origin of LECs in most organs. However, previous reports have not provided sufficient evidence. It still remains to be debated whether LECs are derived from embryonic remnants or pre-existing tissues with acquired inflammatory change.

A unique case with multiple cysts, all of which simulated BCC/LEC microscopically, involving 3 different organs (thyroid gland, parotid gland, and thymus) is reported. It is notable that all of these cysts showed progressive growth in individual unusual organs. In all the previous reports, the authors described a BCC/LEC that arose in a single organ or site. A review of the literature could not identify any report documenting BCCs/IECs that involved multiple organs. This is the first such report, and the histogenesis of the multiple lesions that are quite difficult to interpret is discussed.

## CASE REPORT

A 41-year-old Japanese man was diagnosed as having Hashimoto's thyroiditis in his twenties, and had taken medication for about 20 years. Serological examinations revealed that the titers of antithyroglobulin and antimicrosomal antibodies were continuously greater than 409,600 (normal <100) times. Decreased platelets and elevation of platelet-associated IgG (PA-IgG) were hematologically and sererogically detected since he was 35 years old. Bone marrow puncture was performed, and a diagnosis of idiopathic thrombocytopenic purpura (ITP) was made. The number of platelets in peripheral blood did not recover with steroid administration, eradication of *Helicobacter pyroli*, and splenectomy. Despite these combined therapies, thrombocytopenia remained until the initial left hemithyroidectomy described later. Since then, the platelet number in the peripheral blood has been within the normal range, but the etiology is unclear. The patient suffered from severe pneumonia and pulmonary abscess and was treated by antibiotics at the age of 45 years.

**FIGURE 1:**
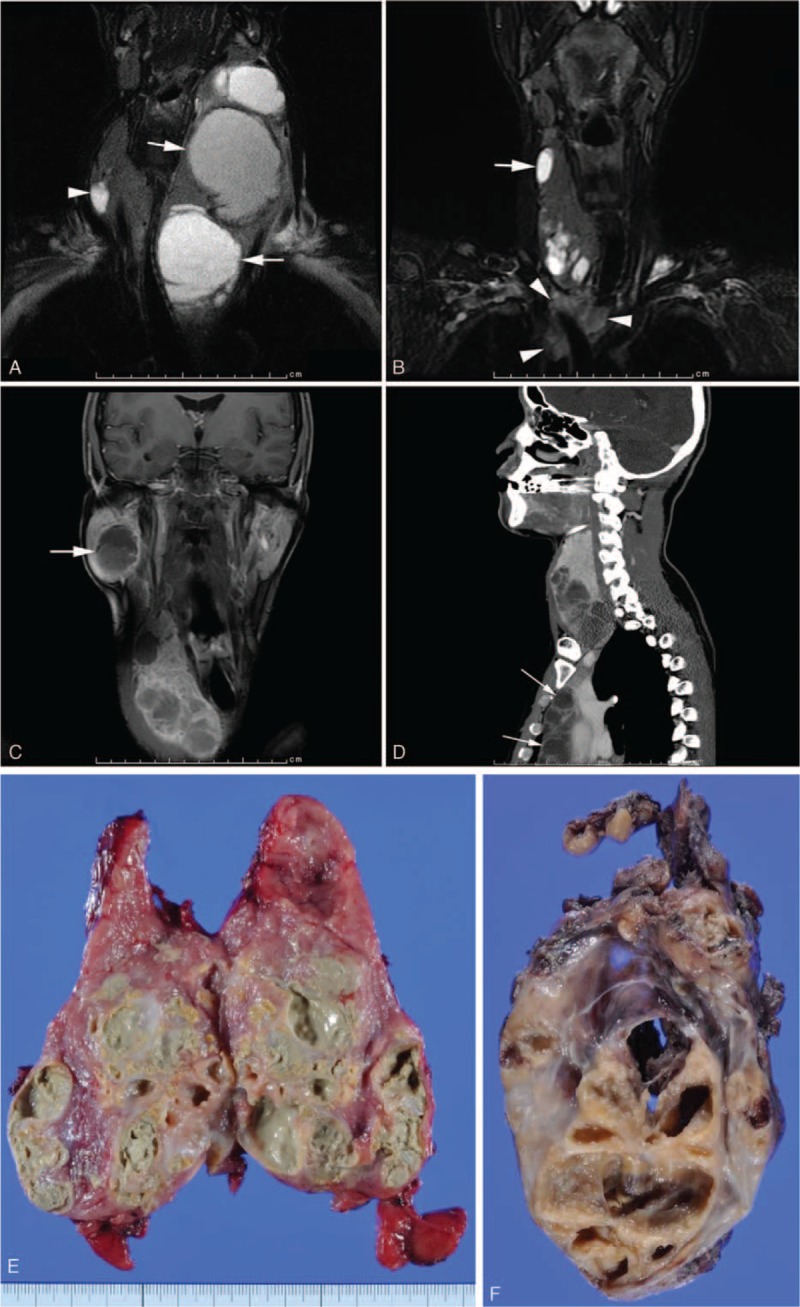
Radiological images (A–D) and macroscopic appearances of the cut surfaces of the cysts (E and F). A, Fat-saturated T2-weighed coronal magnetic resonance (MR) image shows a large multilocular cyst; some of the cystic cavities are more than 5 cm, in the left lobe of the thyroid gland (arrow). A single cyst is also seen in the right lobe (arrow head). B, Fat-saturated T2-weighed coronal MR image demonstrates that the cysts in the right lobe have increased in size and number (arrow). A noncystic lesion has emerged in the superior mediastinum (arrow head). C, Contrast-enhanced T1-weighted MR image. In addition to the pre-existing thyroid cysts, a single cyst has appeared in the right parotid gland (arrow). D, Sagittal reconstructed contrast-enhanced CT shows a multilocular cyst in the superior/anterior mediastinum (arrow). E, A multilocular cyst mainly occupies the lower portion of the thyroid right lobe. The cystic lumens are filled with muddy content. The remainder of the thyroid has reddish to tan color. F, A similar multilocular cyst is seen in the mediastinotomy specimen.

Regarding the mass lesion, the patient noticed an asymptomatic right-sided neck mass showing slow growth since he was 35 years old. Radiological examination revealed that a large multicystic lesion was located in the left lobe of the thyroid gland. In addition, small cysts were dispersed in the right lobe, and a solitary mass was also seen in the right parotid gland (Fig. 1A). A left hemithyroidectomy was performed. A contralateral neck mass appeared and gradually grew after surgery. Radiological images revealed that the pre-existing lesion in the right lobe of the thyroid gland increased in size with deviation of the trachea, and a new lesion emerged in the upper anterior mediastinum (Fig. 1B). These lesions grew up to 10 cm in size (Fig. 1C and D). The mass in the right parotid gland also displayed slight growth (Fig. 1C). A right parotidectomy was performed at the age of 47 years. A right hemithyroidectomy and an anterior mediastinotomy were performed when he was 48 years old.

At present, the patient is healthy with administration of levothyroxine sodium hydrate, and he is being routinely followed for usual interstitial pneumonia, the status of which has been stable for the last 4 years without medication. No mass lesion has been detected for 11 years after the initial left hemi-thyroidectomy.

## METHODS

All sections were cut into 4-μm-thick sections from routinely processed, formalin-fixed, and paraffin-embedded blocks and stained with hematoxylin and eosin (HE) and Alcian blue/periodic acid-Schiff (PAS) staining. Serial sections were submitted to immunohistochemistry and in situ hybridization.

Immunohistochemistry was performed using primary antibodies against thyroid transcription factor-1 (TTF-1; clone 8G7G3/1, Invitrogen, Carlsbad, CA), CD3 (polyclonal, DAKO, Glostrup, Denmark), CD20 (clone L26, DAKO), IgG4 (Zymed Laboratory, San Francisco, CA), thyroglobulin (polyclonal, DAKO), and calcitonin (polyclonal, DAKO) as previously reported.^8^

In situ hybridization was carried out according to the manufacturer's instructions using the GenPoint System (DAKO) with biotylated DNA probes as follows: human papillomavirus (wide-spectrum), adenovirus, cytomegalovirus, Epstein–Barr virus, herpes simplex virus, and SV40 virus (DAKO). Detection of hybridized probe was carried out with primary peroxidase-conjugated streptavidin and biotynil–tyramide secondary peroxidase conjugated streptavidin, and visualized by 3’,3’-diaminobenzidine tetrahydrochloride (Sigma, St Louis, MO). Finally, counterstaining with hematoxylin was performed.

The patient signed informed consent for the publication of case report and accompanying images. The ethical approval was waived by the Yamanashi University ethics committee because this study is a single-case report.

## PATHOLOGIC FINDINGS

### Left Hemi-Thyroidectomy Specimen

Grossly, the left lobe of the thyroid gland measured 5 × 3.5 × 2 cm. On the cut surface, most of the thyroid parenchyma was replaced by a multilocular cyst. Aggregates of small protrusions were found in cystic lumens.

**FIGURE 2:**
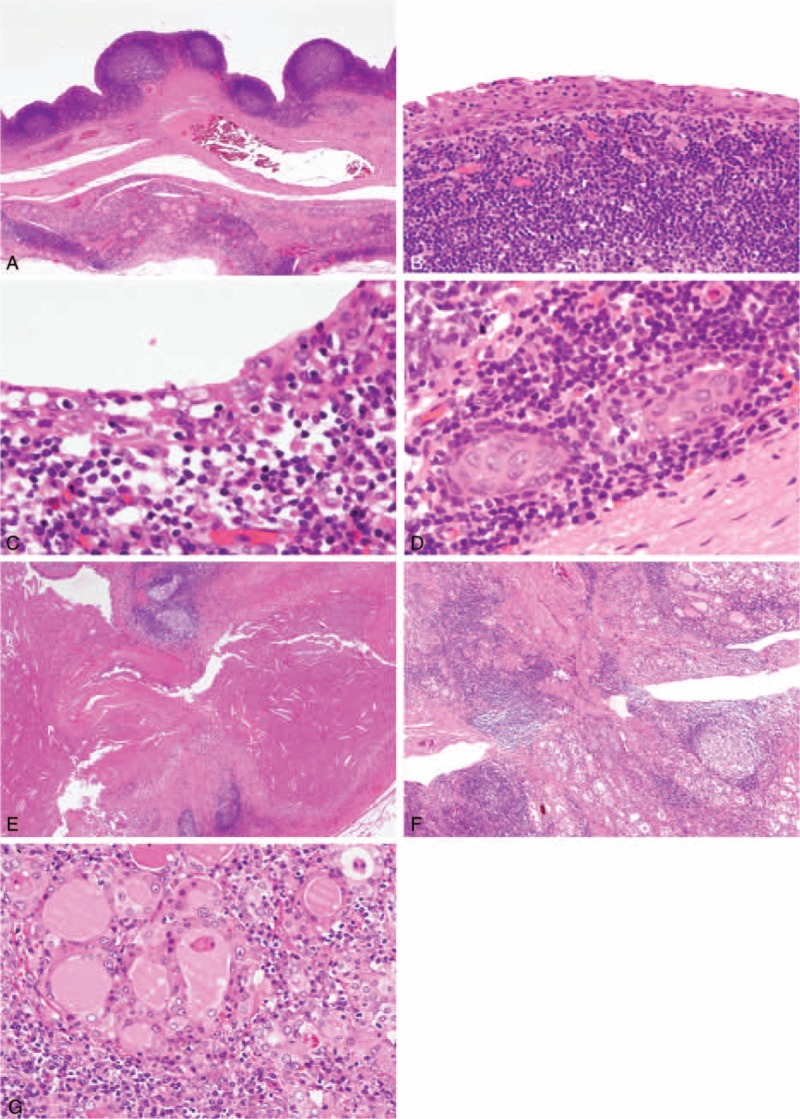
Microscopic findings of the thyroid gland. A, The cyst walls consist of abundant lymphoid tissue and fibrous tissue. Lymphoid follicles protrude into the cystic lumen. B, Most cyst-lining cells are squamous epithelium. C, The cyst lining cells occasionally contain goblet cells. D, Nests of follicular cells with squamous metaplasia are dispersed in the lymphoid tissues. E, Some cystic cavities contain dense eosinophilic material with cholesterin clefts. In the noncystic areas, there is dense lymphocyte infiltration with germinal centers, fibrosis, and dilatation of lymph vessels (F) and eosinophilic change of follicular cells (G).

Microscopically, dense lymphoid tissues with germinal centers were observed beneath lining epithelium (Fig. 2A). The lining cells consisted mainly of single- to several-layered, stratified squamous epitheliums (Fig. 2B). Mucous cells were scattered with reactivity of intracytoplasmic mucin to Alcian-blue/PAS staining (Fig. 2C). The lining cells entirely lacked cilia. Small nests of follicular cells with squamous metaplasia were scattered in the lymphoid tissue of the cyst walls (Fig. 2D). No immature cell nests mimicking solid cell nests (SCNs) were seen throughout the thyroid gland. An outer layer of the cyst wall was composed of fibrous tissue. In some cysts, granulation tissues were formed with desquamation of the lining cells. There were thyroid follicles between the individual cysts. Dense eosinophilic material with cholesterin-clefts was included in some cysts (Fig. 2E). In the background of the thyroid gland, there was dense lymphocyte infiltration with germinal centers, fibrosis, and dilatation of lymph vessels (Fig. 2F) and eosinophilic change of follicular cells (Fig. 2G).

**FIGURE 3:**
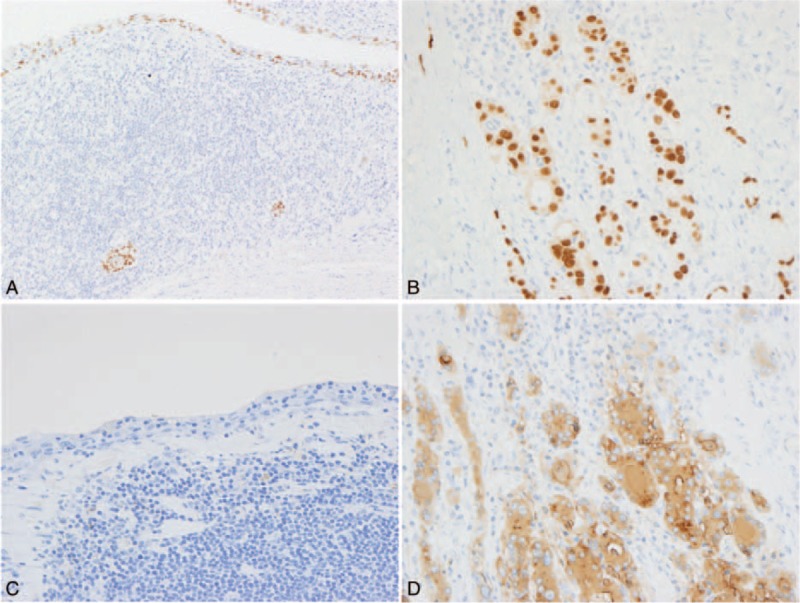
Immunohistochemical results of the thyroid gland. A, The cyst-lining cells and the cells with squamous metaplasia in small nests are positive for thyroid transcription factor-1 (TTF-1). B, Follicular cells are positive for TTF-1. Thyroglobulin reactivity is not detected in the cyst-lining epithelium (C), while it was diffusely observed in both the cytoplasm of the follicular cells and the colloid material (D).

On immunohistochemistry, TTF-1 expression was observed in the cyst-lining epithelium and nests with squamous metaplasia (Fig. 3A), along with follicular cells in noncystic areas (Fig. 3B). Squamous epithelium in the cyst wall was negative for thyroglobulin (Fig. 3C), while follicular cells and colloid material were positive in the background of the thyroid gland (Fig. 3D). A scant amount of calcitonin-positive cells was observed, but the positive cells were not distributed around the cysts.

### Right Hemi-Thyroidectomy Specimen

Grossly, the right lobe of the thyroid gland measured 12 × 7 × 6 cm. A multilocular cyst, 7 × 6 × 6 cm in size, mainly occupied the lower portion of the right lobe (Fig. 1E). Turbid and muddy content filled the cystic spaces. The cut surface except for the cystic lesions had reddish to tan color.

Microscopic findings in both cystic and noncystic areas were essentially identical to those observed in the initial right hemithyroidectomy specimen.

### Anterior Mediastinotomy Specimen

A multilocular cyst measured 14 × 12 × 9 cm. Macroscopic appearances of the cut surface resembled those in the right hemithyroidectomy specimen (Fig. 1F).

**FIGURE 4:**
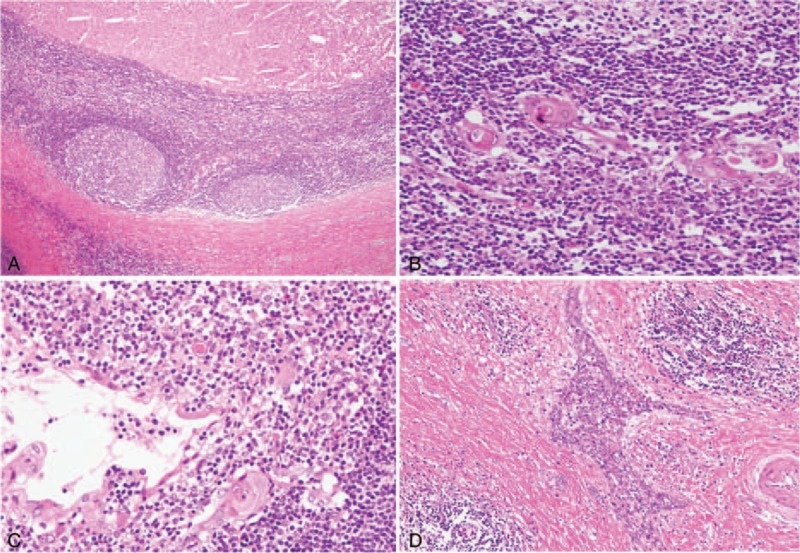
Microscopic finding of the thymus. A, The cysts consist of squamous epithelium, dense lymphoid tissues with germinal centers, and fibrous capsules, including degenerative tissues with cholesterin-clefts. B, Hassal's corpuscles are seen in the lymphoid tissue. C, The corpuscles occasionally show continuity with the cyst-lining cells. D, Islands of thymic epithelium are scattered in the fibrous tissues, showing a branching appearance.

Microscopically, most features were similar to those in the thyroid gland (Fig. 4A). Lymphoid tissues of the cyst walls contained Hassal's corpuscles (Fig. 4B), some of which were continuous with the cyst-lining epithelium (Fig. 4C). Branching islands of thymic epithelium surrounded by fibrous tissue were dispersed (Fig. 4D).

### Parotidectomy Specimen

Grossly, a unilocular cyst, 4.5 × 3.2 cm in size, was observed in the right parotid gland. The cystic space was distended with muddy content.

**FIGURE 5:**
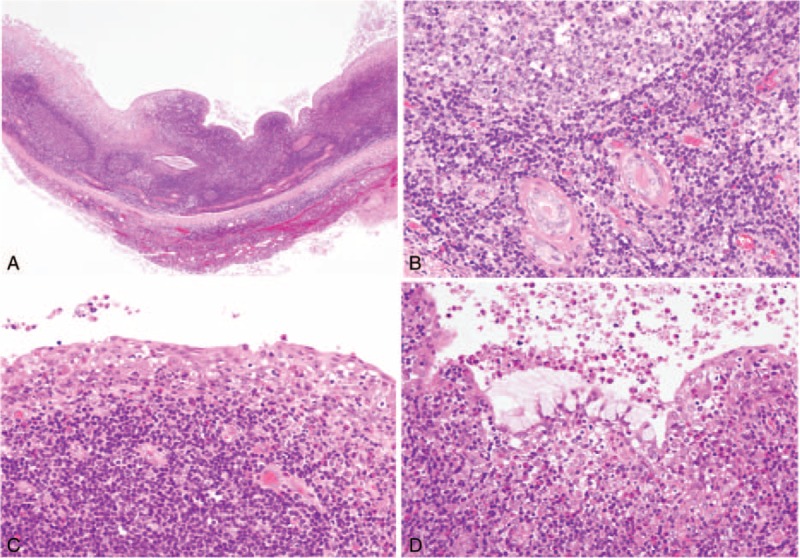
Microscopic findings of the parotid gland. A, The cyst demonstrates features similar to those of the thyroid and thymus at low magnifications. B, Lymphoid tissues include ductal epithelium arranged in small nests and ducts. The cyst-lining cells are mainly composed of squamous epithelium (C) and occasionally of mucous cells without cilia (D).

The microscopic features of the cysts were similar to those in the thyroid gland and thymus (Fig. 5A). In the lymphoid tissue, epithelium arranged in small nests or ducts was scattered (Fig. 5B), showing occasional eosinophilic change. Most lining cells were squamous epithelium (Fig. 5C), and some were mucous cells without cilia showing reactivity for Alcian blue/PAS staining (Fig. 5D).

### Common Findings of the Thyroid Gland, Thymus, and Parotid Gland

Neither monotonous lymphocyte proliferation nor clonal distribution of CD3- and CD20-positive cells was observed. There was no increase of IgG4-positive plasma cells. In situ hybridization was negative for human papillomavirus, adenovirus, cytomegalovirus, Epstein–Barr, herpes simplex, and SV40 virus.

## DISCUSSION

Branchial cleft cysts (BCCs) and lymphoepithelial cysts (LECs) share similar microscopic features. In general, it is dogma that BCCs are derived from embryonic branchial pouch remnants.^1,2^ LECs are rarely located at unusual sites or organs.^3–7^ Based on their morphological similarities, LECs arising from most unusual sites have also been thought to be derivatives of embryonic tissues. In regard to solitary BCC/LECs in a single organ, the hypothesis is reasonable. However, the present patient harbored multiple cysts in multiple organs with clinical complications: Hashimoto's thyroiditis and idiopathic thrombocytopenic purpura (ITP). Therefore, different etiologies may have played roles in the occurrence and development of the lesions.

In the thyroid gland, most cysts result from degeneration of adenomatous goiter. Except for the condition, cystic lesions are extremely rare and include thyroglossal duct cyst, cystic thymic rest, squamous cyst, and branchial cleft cyst/lymphoepithelial cyst (BCC/LEC).^9^ About 25 cases of thyroidal BCC/LEC have been reported to date.^6,7,10–23^ Most intrathyroidal BCC/LECs were unilateral and solitary, but some were bilateral and multiple. From the clinical perspective, the present had Hashimoto's thyroiditis for a long time. Chronic lymphocytic thyroiditis was microscopically observed in the background of the thyroid parenchyma in both hemithyroidectomy specimens. These findings were consistent with Hashimoto's thyroiditis. Interestingly, previous reports indicated that multiple, bilateral, and large-sized BCC/LECs tended to be seen in patients with Hashimoto's thyroiditis and/or chronic lymphocytic thyroiditis.^7,16^ Likewise, in the present case, such long-standing inflammation seems to contribute to the multiplicity and/or growth of the thyroidal cysts.

BCCs/LECs in the thyroid gland have been assumed to originate from embryonic remnants. Some investigators reported that solid cell nests (SCNs) and parafollicular cells (C cells), both of which are derivatives of the ultimobranchial body, were frequently encountered in the vicinity of the cysts.^6^ In addition, respiratory-type epithelium may support the notion that embryonic rests are associated with the histogenesis of the cysts.^6,21^ As previously reported, the speculation is convincing with respect to a thyroidal cyst that presents as a solitary cyst. However, the present patient obviously had different clinical presentations: bilaterality, large size, and multiplicity. Moreover, SCNs were not observed microscopically. Immunohistochemical examinations showed that the cyst walls lacked calcitonin-positive cells in the vicinity of the cysts, and that the cyst-lining epithelium was partially positive for TTF-1. Few mucous cells in the lining epithelium can raise the possibility that thyroidal cysts are of embryonic origin. However, these microscopic features and immunohistochemical results imply that the epithelial component of the cysts originated from the pre-existing thyroid follicular cells showing metaplastic change, rather than embryonic rests.

In the thymus, cysts are also relatively rare, and similar cystic lesions designated as BCCs/LECs have not been reported to date. In 1991, Suster et al first reported the largest series (18 cases) of multilocular thymic cysts (MTCs).^24^ According to this report, the histopathological features coincided with those seen in the present case.^24^ Thymic epithelial tumors, such as thymoma and basaloid carcinoma, may coexist in MTCs.^24,25^ In this case, extensive microscopic examination using many sections failed to disclose any neoplasms including malignant lymphoma. Neither embryonic remnant nor ectopic tissues were obviously identified. Moreover, the epithelial component was thoroughly negative for thyroid transcription factor-1 and thyroglobulin. Interestingly, the patients with the MTC often had immune and/or hematopoietic disorders: Sjögren syndrome, aplastic anemia, chronic myeloid leukemia, Duncan syndrome (X-linked lymphoproliferative disease), and acquired immunodeficiency syndrome.^24^ In particular, Sjögren syndrome seems to have a strong correlation with MTCs.^26,27^ The frequent presence of these diseases or disorders may imply that such immunological abnormalities play a role in the occurrence and/or growth of MTCs.

BCCs/LECs occasionally occur in a parotid gland. An embryonic origin has not been regarded as the pathogenic process of the lesion arising from this organ. The lesions have been thought to originate from an intraparotid gland lymph node^28^ or lymphofollicular hyperplasia^29^ or Sjögren syndrome-like conditions associated with human immunodeficiency virus (HIV).^30^ However, the origin of BCCs/LECs in the parotid gland still remains to be determined. It has been well known since the 1990s that some counterparts of BCCs/LECs are closely associated with HIV. The HIV-associated BCCs are more inclined to be bilateral, multiple, and accompanied by neck lymphadenopathy^4,29,31^ than BCCs in HIV-negative patients.^32^ The patient clinically presented neither HIV infection nor neck lymphadenopathy, and the BCC was solitary and unilateral.

Regarding the histogenesis of multiple cysts, viral infection was considered, but in situ hybridization failed to detect integration of representative viruses in all resected specimens. Moreover, increased levels of IgG4-positive cells were not detected, suggesting that the lesions were not IgG4-related disease. It is difficult to clarify their actual etiology based upon this single-case report. Further accumulation of similar cases will be necessary.

In summary, this case is unusual in that multiple cysts, all of which showed BCC/LEC-like features, arose in 3 different organs: thyroid gland, parotid gland, and thymus. The lesions synchronously and/or heterochronously showed progressive growth in the individual organs. No similar cases having such multiple cysts that occurred in different organs could be identified in the literature. From a clinical perspective, the patient had autoimmune disease (Hashimoto's thyroiditis) and ITP. Moreover, he had suffered from a severe respiratory infection, despite his young age and no evidence of being immunocompromised. It is difficult to exclude the possibility that all of the lesions arose incidentally and/or independently in different organs. Nevertheless, common histopathological features, along with characteristic clinical complications, might imply that systemic immune and/or hematopoietic disorders may underlie their generation and development in conjunction with putative genetic alterations.
